# Impact of an ultrasound-driven diagnostic protocol at early intensive-care stay: a randomized-controlled trial

**DOI:** 10.1186/s13089-019-0139-2

**Published:** 2019-09-30

**Authors:** Julio Pontet, Christian Yic, José L. Díaz-Gómez, Pablo Rodriguez, Igor Sviridenko, Diego Méndez, Sylvia Noveri, Ana Soca, Mario Cancela

**Affiliations:** 1 Intensive Care Unit, Hospital Pasteur, Administración de Servicios de Salud del Estado, Montevideo, Uruguay; 2 Intensive Care Unit, Asociación Española Primera de Socorros Mutuos, Montevideo, Uruguay; 3 Department of Critical Care Medicine, Mayo Clinic, Jacksonville, USA; 4 Larravide 2458, Montevideo, Uruguay

**Keywords:** Clinical impact, Critical care, Diagnostic techniques and procedures, Point-of-care ultrasound, Point-of-care systems, Ultrasound protocol

## Abstract

**Background:**

Point-of-care ultrasound (POCUS) is a tool in increasing use, but there is still a lack of basics for its routine use and evidence of its impact in intensive care.

**Objective:**

To measure the impact of POCUS on resource utilization, diagnostic accuracy, and clinical management in medical-surgical intensive care units (ICUs).

**Methods:**

Prospective, controlled study, in two polyvalent ICUs. The patients were randomly assigned to POCUS or control group.

**Interventions:**

POCUS patients received systematic ultrasound examination of optic nerve, lung/pleura, heart, abdomen, and venous system, performed at the bedside by trained intensivists. Control patients were treated by critical care specialists who do not perform ultrasound in their clinical practice.

**Results:**

We included 80 patients, 40 per group. There were no significant differences in age, sex, APACHE II score, or admission diagnosis. POCUS group used fewer resources per patient in the first 5 days of hospitalization: chest radiography (2.6 ± 2.0 vs 4.1 ± 3.5, *P *= 0.01), additional ultrasound evaluations performed by a radiology specialist (0.6 ± 0.7 vs 1.1 ± 0.7, *P *= 0.002), and computed tomography studies (0.5 ± 0.6 vs 0.9 ± 0.7, *P *= 0.007). Time to perform any requested ultrasound evaluation after ICU admission was 2.1 ± 1.6 h versus 7.7 ± 6.7 h (*P *= 0.001). Systematic ultrasound evaluation led to better characterization of ICU admission diagnosis in 14 (35%) patients and change in clinical management in 24 (60%). POCUS group had lower fluid balance at 48 and 96 h after admission (*P *= 0.01) and spent less time mechanically ventilated (5.1 ± 5.7 days vs 8.8 ± 9.4, *P *= 0.03).

**Conclusions:**

Systematic application of POCUS may decrease utilization of conventional diagnostic imaging resources and time of mechanical ventilation, and facilitate meticulous intravenous fluid administration in critically ill patients during the first week of stay in the ICU.

*Trial registration* ClinicalTrials.gov Identifier: NCT03608202.

## Introduction

Ultrasonography is currently considered almost essential in the management of patients in shock, acute respiratory failure, or multiorgan dysfunction [1–4]. There is still a broad field of research aimed at generating a transformational change based on scientific evidence in critical care ultrasound [5, 6]. Given the inherent limitations for an accurate physical examination of the critically ill, imaging modalities such as chest, abdominal radiography, and computed tomography (CT) are considered more reliable, useful, and impactful in the critical care setting. However, their portability, versatility, and costs are less than ideal [1], with unnecessary delays in obtaining a diagnosis in the critically ill, and increased risk for hypoxemia or refractory arterial hypotension during transportation [3]. In contrast, ultrasound, a widely available, simpler technique that can be performed with portable equipment at the bedside, has gained progressive acceptance [4].

Recent investigations have demonstrated that multiorganic ultrasound decreases resource utilization and guides clinical management of critical ill patients while enhancing diagnostic accuracy [7–11]. Nevertheless, these previous publications lack a randomized and controlled trial methodology. We hypothesize that routine use of methodic, multisystemic ultrasound protocol in critically ill patients at admission to the intensive-care unit (ICU) reduces utilization of diagnostic resources and consultation and time to definitive diagnosis and tracheal extubation. Thus, the aims of our investigation were to analyze diagnostic and therapeutic implications of a point-of-care ultrasound (POCUS)-driven protocol during the first 5 days of ICU admission.

## Materials and methods

### Study design

Our prospective, randomized, controlled study was conducted at two major referral hospitals, Asociación Española Primera de Socorros Mutuos and Hospital Pasteur, Administración de Servicios de Salud del Estado. Patients admitted to a 12-bed medical-surgical ICU and 8-bed stepdown unit in those institutions were included in the study. The study protocol was approved by the Institutional Ethics Committee, and informed consent was obtained prior to each patient’s enrollment.

### Patients

We screened patients between February 1, 2017 and May 31, 2017, admitted to the ICUs. Inclusion criteria to the study were: (1) 18 years of age or older; (2) mechanical ventilation (MV) required at ICU admission. Patients with more than 24 h of hospitalization and those considered with low likelihood of survival by the treating intensivist at ICU admission were excluded.

### Clinical data

We collected pertinent clinical data, including Acute Physiology and Chronic Health Evaluation, second version (APACHE II) score at admission, reason for admission to the ICU, and other relevant physiologic data.

The Distensibility index of inferior vena cava (%) was utilized as the primary measure for fluid responsiveness based on a previous investigation [12] (Table 1). In addition, we routinely used invasive MAP monitoring, capillary filling, hourly diuresis, central venous saturation, arteriovenous CO_2_ difference, and plasma lactate measurement alongside measure of cardiac output and LVEF as supportive parameters of the clinical-decision making of administering intravenous fluids to patients suffering from shock.

**Table 1:** POCUS protocol

Type of ultrasound	Instruments questions	Theoretic considerations
Optical behavior	Diameter optic nerve: right eye (mm); left eye (mm)	A diameter > 5.7 is a noninvasive indication of intracranial hypertension
Neck anatomy	Visualization of great vessels (jugular vein, carotid artery): normal or abnormal	Detection of patency (thrombosis) and anatomical variants or abnormalities
Pulmonary	Lung ultrasound score, 0–36 points	Score increases as pulmonary water increases; indicates pulmonary edema
Pleural	Presence of pleural occupation and estimation of pleural effusion (mL)	Confirmation of diagnosis, volume evaluation and follow-up
Echocardiography overall function	Estimation of left-ventricular systolic function by LVEF (%) and right-ventricular systolic function by TAPSE (mm)	LVEF > 50% and TAPSE > 15 mm is considered normal
Prediction of volume responsiveness	Distensibility index of inferior vena cava (%)	An index > 12% indicates response to intravenous fluid challenges
Estimation of CO	CO (L/min) estimated by left ventricular outflow tract by velocity time integral and diameter	Normal values, 4–6 L/min
Abdominal screening	FAST protocol for presence or absence of intraperitoneal free fluid	Presence of free fluid is abnormal
Biliary	Presence of lithiasis or dilated biliary tract	Biliary tract pathology may be an incidental finding or the cause of critical illness
Renal	Presence of urinary lithiasis or dilated urinary tract	Urinary tract pathology may be an incidental finding or the cause of critical illness
Ultrasound-guided invasive procedures	Venous or arterial access, pleural or abdominal drainage	Ultrasound-guided invasive procedures are more secure, with fewer adverse effects

*CO* cardiac output, *FAST* focused assessment with sonography in trauma, *IH* intracranial hypertension, *LVEF* left-ventricular ejection fraction, *POCUS* point-of-care ultrasound, *TAPSE* tricuspid annular plane systolic excursion

### Intervention

Two groups were compared; the study group received routine evaluation with an ultrasound-driven protocol (POCUS group), while a control group received conventional management according to the pre-established protocols in the ICU (in sepsis, pneumonia, postoperative care, heart failure and myocardial ischemia, trauma, COPD, etc.).

Patients were randomly assigned by permuted blocks to either the POCUS group or the control group.

The POCUS protocol is shown in Table 1; the following variables were evaluated: confirmation of initial diagnosis; change in initial diagnosis; new unsuspected finding; lack of change in initial diagnosis, inability to rule out a condition, and no changes in treatment; association with wrong diagnosis; and association with subsequent pharmacologic, medical, procedural, or surgical measures. In addition, these resource- and outcome-related variables were analyzed for comparison between the two groups: number of additional radiologic, ultrasound, and CT studies; number and type of procedures or interventions ordered; time to for ultrasound evaluation; duration of MV; length of stay in the ICU; and mortality rate.

POCUS studies were conducted with Logiq-e (General Electric Healthcare Japan Corporation) system with digital image storage capacity. Clinical data were saved in the SPSS Statistics 23 program (International Business Machines Corp.).

### Statistical analysis

With a sample size of 40 patients per group for an annual population of 400 patients admitted to each ICU, we estimated a margin of error of 14% (95% CI). The qualitative variables were records as percentages and the quantitative as mean ± standard deviation. For the comparison of mean between groups, either the Student’s *t* test for independent groups or Mann–Whitney test was applied according to the case; and binaries in contingency tables by χ^2^ or Fisher’s exact test. A *P* value < 0.05 was considered significant in all tests.

## Results

### Patient demographics

We included 80 patients, 40 in each group. In the POCUS group, 24 were men (61%) and 16 were women (39%); similarly, in the control group, there were 25 men (62%) and 15 women (38%). There were no statistical differences between the groups regarding age, sex, APACHE II score at 24 h after admission, or reason for admission to ICU (Table 2).

**Table 2:** Demographic data and diagnosis at admission in each group

Groups (*N* = 80)	POCUS (*n* = 40)	Control (*n* = 40)	*P* value
Age, median ± SD, y	60 ± 15	57 ± 15	0.99
Sex, no. (%), male	24 (60.0)	23 (57.5%)	0.99
APACHE II, median ± SD	27 ± 9	26 ± 7	0.99
Diagnosis at admission, no. (%)			0.99
Respiratory sepsis	6 (15.0)	4 (10.0)	
Severe community-acquired pneumonia	5 (12.5)	3 (7.5)	
Decompensated heart failure	5 (12.5)	4 (10.0)	
Peritoneal sepsis	4 (10.0)	4 (10.0)	
Pulmonary tuberculosis	2 (5.0)	1 (2.5)	
Guillain–Barré syndrome	2 (5.0)	1 (2.5)	
Sepsis unknown origin	1 (2.5)	0 (0.0)	
Third-degree AV block	1 (2.5)	2 (5.0)	
Stroke	3 (7.5)	3 (7.5)	
COPD exacerbation	2 (5.0)	5 (12.5)	
Acute myocardial infarction	2 (5.0)	2 (5.0)	
Severe trauma	2 (5.0)	3 (7.5)	
Suicide attempt	2 (5.0)	3 (7.5)	
Acute bacterial meningitis	1 (2.5)	0 (0.0)	
Pulmonary embolism	1 (2.5)	1 (2.5)	
Thyrotoxicosis	1 (2.5)	0 (0.0)	
Enteric sepsis	0 (0.0)	2 (5.0)	
Acute encephalitis	0 (0.0)	1 (2.5)	
Status epilepticus	0 (0.0)	1 (2.5)	

*APACHE II* Acute Physiology and Chronic Health Evaluation II, *AV* atrioventricular, *COPD* chronic obstructive pulmonary disease, *POCUS* point-of-care ultrasound

### Clinical information or decision-making following POCUS

The POCUS protocol was performed in 15–30 min in all cases.

In the POCUS group, 48 clinical decisions were made in 36 of 40 patients (90%). Furthermore, 35% of these observations were related to the diagnostic category, and either due to new or changed diagnosis. Modifications in pharmacologic therapy occurred in 24 patients (60%), and invasive maneuvers or procedures were necessary in 9 (23%) (Table 3).

**Table 3:** Description of changes in clinical information or decisions led by ultrasound

Modification in diagnosis and therapeutic decisions led by US	No. changes	No. patients
Related to clinical decision-making, total	48	36
New or unidentified diagnosis: Pneumonia, 2; significant pleural effusion, 5; pneumothorax, 1; significant pericardial effusion, 1; cholecystitis, 1	10	8
Clinical diagnosis: Pneumonia to respiratory distress due to biliary sepsis, 1; pneumonia to heart failure, 2; asthma to pneumonia, 1	4	4
Pharmacological therapy: Fluid challenges, 6; start diuretics, 5; dobutamine, 5; noradrenaline, 2; antibiotics, 5; alteplase, 1	24	16
Invasive procedures: Thoracic drainage, 5; emergency bronchoscopy, 2; laparotomy, 1; suprapubic bladder catheterization, 1	9	7
Alveolar recruitment maneuver, 1	1	1
No changes	0	4

*US* ultrasound

### Utilization of additional diagnostic testing

Overall, there was lower utilization of resources per patient during the first 5 days of ICU stay in the POCUS group compared with the control group (Table 4): lower number of chest radiography requested (2.6 ± 2.0 vs 4.1 ± 3.5, respectively, *P *= 0.01); lower utilization of additional ultrasound ICU imaging per radiology specialist (0.6 ± 0.7 vs 1.1 ± 0.7, respectively, *P *= 0.002); and fewer CT scans performed (0.5 ± 0.6 vs 0.9 ± 0.7, respectively, *P *= 0.007).

**Table 4:** Comparison of resource utilization, length of stay and mortality in ultrasound-driven evaluation versus conventional management in ICU

Variable	POCUS group (mean ± SD)	Control group (mean ± SD)	*P* value
Chest radiography^a^	2.6 ± 2.0	4.1 ± 3.5	0.01
US requested outside of ICU^a^	0.6 ± 0.7	1.1 ± 0.7	0.002
Computed tomography^a^	0.5 ± 0.6	0.9 ± 0.7	0.007
Mechanical ventilation, days	5.1 ± .7	8.8 ± 9.4	0.03
Length of stay in ICU, d	9 ± 8	13 ± 10	0.05
Mortality, no. (%)	7 (17)	6 (15)	> 0.99

*Echo* echocardiography, *ICU* intensive-care unit, *US* ultrasound

^a^During the first 5 days of intensive-care unit stay

### Analysis of delay times

We measured time from admission to first ultrasound (delay time). The delay in performing diagnostic ultrasound was significantly reduced in the POCUS group versus control (2.1 ± 1.6 h vs 7.7 ± 6.7, *P *= 0.001). In the control group, all diagnostic ultrasound evaluations were performed by radiology specialists available via telephone.

### Clinical outcomes

#### Fluid management

**Fig. 1:**
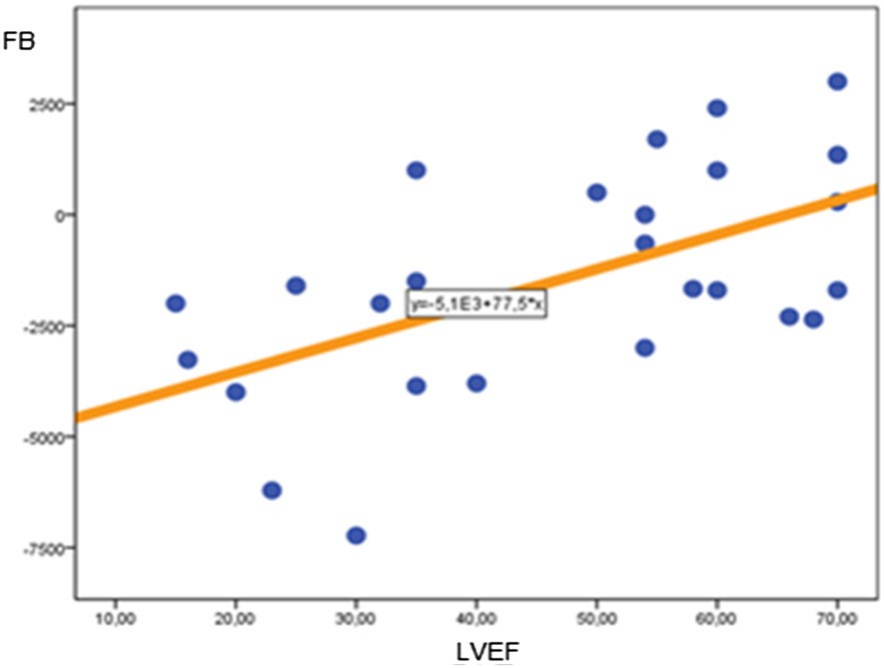
Linear correlation between fluid balance at 48 h and left-ventricular ejection fraction. Pearson correlation *r* = 0.57, *P *= 0.002. *FB* fluid balance, *LVEF* left-ventricular ejection fraction

The POCUS-driven decision of fluid management in each patient was left at treating intensivist discretion. Similarly, clinical decisions on fluid, inotropic, or vasopressor therapy, and adjustments to MV were made based on POCUS assessment. Fluid balance (FB) was much lower in the POCUS group than in the control at 48 and 96 h after ICU admission (− 1600 ± 2750 mL vs − 400 ± 2130 mL, *P *= 0.03, and − 3200 ± 3510 mL vs − 890 ± 3900 mL, *P *= 0.006, respectively). There was a significant positive linear correlation between left-ventricular ejection fraction (LVEF) and FB at 48 (Pearson correlation *r* = 0.57, *P *= 0.002) (Fig. 1) and 96 h (*r* = 0.58, *P *= 0.03).

#### Duration of MV

The POCUS group showed a lower duration of MV than the control (5.1 ± 5.7 days vs 8.8 ± 9.4 days, *P *= 0.03) (Table 4). There was no significant correlation between the time spent on MV and FB, or between the time spent on MV and LVEF.

### Length of stay in ICU and mortality

Mortality rates did not differ between POCUS and control groups. Of note, there was a tendency for decreased ICU length of stay with POCUS (Table 4).

## Discussion

We demonstrated the direct impact of a routine POCUS-driven protocol in patients during the early phase after admission to medical–surgical ICUs. A systematic application of POCUS resulted in decreased utilization of conventional diagnostic imaging resources and time of MV, and facilitated a judicious intravenous fluid administration in critically ill patients during the first week of ICU hospitalization. Application of routine POCUS appears to be safe if utilized by appropriately trained intensivist as no complications were directly associated with POCUS use in the study group.

Previous investigations [2, 5] applying particular ICU-sound protocol led to more accurate admission diagnosis in 25.6% to 24.9%. They had an observational design, but are comparable to the 22% accuracy rate of new or corrected diagnoses in our study. Moreover, the majority of our patient population suffered from sepsis. Manno et al. [2] proposed that septic patients may be the most favored subgroup of patients receiving a POCUS protocol because of the myriad of information and therapies that can be tailored to each individual patient.

The main effect of routine utilization of our POCUS protocol was the clinical decision-making, more specifically in pharmacologic management adjustments in shock patients (60%) and ordering of ultrasound-guided procedures (23%). In our study, we found that 23% of our population required ultrasound-guided invasive procedures, which is similar to the 21.6% reported by the Manno group [2]. Vignon et al. [13] described a change in therapeutic management of patients in 51% of the cases, and Bernier-Jean et al. [5] in 44%.

Management of intravascular volume status can be a difficult decision at the bedside. Meticulous fluid management in treating shock patients with heart disease is desirable. In our case, the most common clinical decision was related to fluid management. Similar findings have been described in an observational study [13], but we emphasize the randomized-controlled design of our study. Nonetheless, our direct comparison of timely ultrasound-driven versus conventional management allows us to represent the current practice in the majority of medical centers due to lack of routine utilization of ultrasound by intensivists. Furthermore, positive fluid responsiveness test does not indicated necessity of further fluid administration, our clinical-decision making was determined by the echocardiography measurements (LVEF, IVC distensibility index) in addition to systemic perfusion surrogates (central venous saturation, arteriovenous CO2 difference, and plasma lactate measurements). Finally, in cases of heart failure, we guided restriction of fluids by signs of pulmonary and systemic congestion. However, we acknowledge tolerance to fluids must be assessed with diastolic function parameters and may be in addition to lung ultrasonography [14–16].

Our POCUS protocol led to a significantly lower utilization of chest radiography, ultrasound performed by non-intensivist specialists, and CT scans (up to 56% reduction in CT requests). We found a reduction of radiology evaluation utilization from 4.1 in the control group to 2.6 in the POCUS group (63% reduction). This finding differs from other groups reporting 22% to 26% reduction in radiography utilization [17, 18]. Possible explanations include intensivist discretion versus specific protocol, considerable critical care ultrasound expertise by attending intensivist at time of the study, and more availability of fully capable ultrasound devices. Of note, the American College of Radiology expert panel recommended daily radiology for each intubated patient [17]. The direct clinical implications of our findings were associated with lower radiation exposure, less intra-hospital transportation of unstable patients, and inherent economic savings with more sophisticated evaluations, including in improved imaging suites workflow and reduced necessity for personnel to transport patients [19–21].

We found a significant correlation between cardiac function (left systolic ventricle function measured by LVEF index) and a negative FB in the first 5 days of ICU management. This might indicate that judicious fluid management and decisions in administration of diuretic agents are facilitated with real-time utilization of POCUS since ICU admission. We did not find a significant correlation between time spent on MV and FB or between duration of MV and LVEF. This can possibly be explained by the heterogeneity of our population. However, there was correlation between lower LVEF and negative FB, since patients with low LVEF are likely to receive less fluids and more diuretics.

We acknowledge several limitations in the current investigation. First, our small patient population avoids finding significant differences in meaningful clinical outcomes, such as mortality. Second, we analyzed only the first 5 days of ICU stay. However, as patients stay longer in the ICU, additional confounding factors not necessarily related with admission diagnosis can affect the outcomes (late-onset, ventilator-associated pneumonia). Mortality was similar between both groups, but it should be noted that future studies with larger patient populations having a shorter time of MV as found in the POCUS group could potentially determine a decrease in mortality related to MV complications. Third, we did not find a significant correlation between normal or high ejection fraction and pulmonary B profile in our study that can imply possible tolerance to fluid administration. However, patients with CHF and preserved EF can be only characterized with advanced echocardiography parameters of diastolic function. We recognize that the lack of diastolic function assessment is another limitation of this study. However, we aimed to evaluate a practical approach for assessment of ventricular function and fluid responsiveness, so we propose further investigation of the correlation between normal or high ejection fraction and pulmonary B profile in addition to diastolic function assessment in septic shock patients.

Finally, the absence of a reposition protocol guided by pre-established ultrasound should be endorsed in multicentric studies.

## Conclusions

A systematically applied POCUS protocol upon admission to the ICU can guide diagnostic and therapeutic decisions in critically ill patients. This potentially leads to greater resourcefulness and shorter duration of MV. Definitive associations, such as ultrasound-driven fluid resuscitation and improved outcomes, remain to be proven in larger multicenter studies. Diastolic function assessment can improve overall evaluation of fluid tolerance to the fluid management of septic shock patients.

## Data Availability

The data sets used and/or analysed during the current study are available from the corresponding author on reasonable request.
